# A Virulence Factor from *Sclerotinia sclerotiorum* Targets the Host Chloroplast Proteins to Promote Infection

**DOI:** 10.3390/plants13233430

**Published:** 2024-12-06

**Authors:** Wenjing Cui, Kunqin Xiao, Feng Yang, Kaibin Qiao, Xun Xu, Songyang Gu, Jinxin Guo, Zhuojian Song, Hongyu Pan, Fengting Wang, Yanhua Zhang, Jinliang Liu

**Affiliations:** College of Plant Sciences, Jilin University, Changchun 130062, China; cuiwenjingjlu@163.com (W.C.); 13756949284@163.com (K.X.); yangfengjlu@163.com (F.Y.); kaibinqiao@163.com (K.Q.); mmxx83132@163.com (X.X.); gusy23@jlu.edu.cn (S.G.); verygjx@163.com (J.G.); jiachuang453@163.com (Z.S.); panhongyu@jlu.edu.cn (H.P.); wft1001@jlu.edu.cn (F.W.); yh_zhang@jlu.edu.cn (Y.Z.)

**Keywords:** *S. sclerotiorum*, chloroplast-targeted protein, Coproporphyrinogen-III oxidase, shikimate kinase 2, plant immunity

## Abstract

Chloroplasts are not only places for photosynthesis, but also participate in plant immunity and are important targets of pathogens. Pathogens secrete chloroplast-targeted proteins (CTPs) that disrupt host immunity and promote infection. *Sclerotinia sclerotiorum* (Lib.) de Bary is a phytopathogenic fungus with a broad host range. However, little is known about the pathogenic mechanisms underlying this wide host range. In this study, we investigated the role of Chloroplast-Targeted Protein 1 (SsCTP1) secreted by *S. sclerotiorum* in pathogenesis, which inhibits plant immunity and promotes pathogen infections. *SsCTP1* was highly up-regulated during the early stages of *S. sclerotiorum* infection in various hosts, and its transient expression in *Nicotiana benthamiana* revealed that it was predominantly localized within chloroplasts. Mutants with *SsCTP1* deletion exhibited a similar growth rate and colony morphology to the wild type, but significantly reduced pathogenicity in various hosts. Moreover, SsCTP1 inhibited chitin-induced callose deposition and defense gene expression, and enhanced sensitivity to *S. sclerotiorum* in *N. benthamiana*. Similarly, transgenic *Arabidopsis thaliana* overexpressing SsCTP1 displayed an increased susceptibility to *S. sclerotiorum*. Furthermore, two host proteins that interact with SsCTP1, Coproporphyrinogen-III oxidase (GmCPX), and shikimate kinase 2 (GmSKL2) were identified by screening the soybean cDNA library, and these interactions were confirmed in vivo. Importantly, the silencing of *NbCPX* by virus-induced gene silencing enhanced *N. benthamiana* resistance to *S. sclerotiorum*. Our results indicate that SsCTP1 is an important pathogenic factor that contributes to the wide host range of *S. sclerotiorum* and may inhibit plant immunity by targeting the chloroplast proteins GmCPX and GmSKL2, which are ubiquitous in host plants.

## 1. Introduction

As guardians of their own defense, plants employ intricate systems to perceive pathogenic threats. Plasma membrane-resident pattern recognition receptors (PRRs) identify pathogen-associated molecular patterns (PAMPs), initiating a foundational immune response known as PAMP-triggered immunity (PTI). Concurrently, within the cell, nucleotide-binding leucine-rich repeat domain-containing receptors (NLRs) interact with pathogen effectors, instigating a heightened immune reaction called effector-triggered immunity (ETI) [1,2,3,4]. These two immune pathways, PTI and ETI, are not mutually exclusive; rather, they integrate their signals to orchestrate a comprehensive and potent defense mechanism in plants [5].

A two-stage infection model of *S. sclerotiorum* has been established based on cytological and genetic evidence. Initially, the fungus releases effector molecules designed to suppress or interfere with the host plant immune response. Subsequently, it secretes oxalic acid (OA) [6] and cell wall degrading enzymes (CWDEs) [7,8], which contribute to the demise of plant cells. This model elucidates the pathogen’s strategy for overcoming plant defenses, facilitating its colonization and spread within the host tissue [9]. In most cases, necrotrophic pathogens such as *Botrytis cinerea* and *S. sclerotiorum* proliferate in dead tissues and secrete effectors that induce host cell necrosis. *Verticillium dahliae* effectors VdEG1 and VdEG3 require SOBIR1 and BAK1, respectively, to elicit immunity in *N. benthamiana* [10]. The effector protein Ecp6 from *Cladosporium fulvum*, a LysM domain-containing chitin-binding protein, competes with the host LysM receptor for chitin binding in the apoplast, thereby suppressing chitin-triggered immune responses [11]. The chitinase MoChia1 from *Magnaporthe oryzae* activates immune responses in rice apoplasts. MoChia1 binds to chitin and inhibits the chitin-induced ROS burst in rice [12].

A small secreted protein rich in cysteine, SsSSVP1, internalizes in plant cells and hijacks the mitochondrial protein QCR8 into the cytoplasm to disrupt its normal functional localization, leading to plant cell death [13]. The apoplastic effector protein SsCP1 induces host cell death and targets PR1 to suppress its antimicrobial activity, thus facilitating *S. sclerotiorum* infection [14]. Additionally, the receptor protein for SsCP1 on the plant cell membrane can be recognized, initiating a defense response dependent on the SA signaling pathway. The *S. sclerotiorum* effector SsITL directly targets the calcium sensing receptor (CAS) on chloroplasts to inhibit SA accumulation in the host plant during the early stages of infection, thereby suppressing host immunity [15]. Moreover, secreted proteins, such as SsCaf1, SsRhs1, and SsERP1, have also been reported to be involved in the pathogenicity of *S. sclerotiorum* [16,17,18].

Coproporphyrinogen-III oxidase (CPX) is a pivotal enzyme in the biosynthesis of tetrapyrroles that catalyzes the final conversion of coproporphyrinogen to protoporphyrin IX. The tetrapyrrole biosynthetic pathway occurs within chloroplasts and is crucial for the fine regulation of plant growth, development, and environmental adaptation [19,20]. Tetrapyrroles play a multitude of vital roles in various biological processes, including light harvesting, photophosphorylation, reactive oxygen species (ROS) scavenging, and oxygen transport [20]. Studies have indicated that *LIN2* encodes coproporphyrinogen III oxidase (CPX), an enzyme that plays a crucial role in tetrapyrrole biosynthesis. The rice photo-oxidation leaf damage mimic mutant 1 (*llm1*) exhibits lesion mottling in leaves during the tillering stage [21,22]. The formation of lesion spots in the *llm1* mutant is attributed to programmed cell death and ROS production. Under conditions of increased expression of pathogenesis-related genes (PRs), the *llm1* mutant shows enhanced resistance to bacterial wilt pathogens. Disruption of *CPX* leads to the formation of necrotic spots [23,24,25]; *GmLMM2* encodes CPX in the chloroplasts, and suppression of *GmLMM2* disrupts the chloroplast structure and the tetrapyrrole synthesis pathway [26].

Shikimate kinase is an enzyme that catalyzes the fifth step of the shikimate pathway, which involves the conversion of shikimate to shikimate-3-phosphate. The shikimate pathway is an important metabolic pathway in all living organisms. The shikimate metabolic pathway converts carbon metabolism in green plants into aromatic compounds, providing substrates for other secondary metabolic pathways. After the metabolic processes, metabolites act as signaling molecules to regulate the growth and development of plants and endow them with the ability to resist pests and diseases [27]. In rice, OsSKL2 can interact with OsASR1, and, together, they act as important regulatory factors, conferring oxidative stress resistance as well as salt and drought tolerance by scavenging ROS [28]. In *Arabidopsis,* SKL1 affects chloroplast biogenesis by regulating the auxin pathway [29], whereas *AtSK1* and *AtSK2* are expressed bidirectionally under biotic and abiotic stress conditions [30]. The overexpression of *ZmSKL1* and *ZmSKL2* genes in *A. thaliana* can enhance plant tolerance to drought stress. This suggests that *SKL* genes may enhance plant resilience to stress by regulating water retention mechanisms in plants [31]. The co-expression of OsSKL2 and OsASR1 can reduce ROS accumulation, suggesting that these two proteins may work together to maintain the REDOX balance in plants under stress conditions. These findings not only reveal novel functions of SKL2 in plant immunity and stress responses, but also provide potential molecular targets for the development of crop varieties with enhanced stress tolerance. Future studies should further explore the interactions between SKL2 and other immune-related genes, as well as the specific mechanisms of action within the plant immune network.

Here, we elucidated the mechanism of the chloroplast-targeted effector, SsCTP1, in the omnivorous pathogenesis of *S. sclerotiorum*. Genetic evidence has revealed that SsCTP1 is crucial for the *S. sclerotiorum* infection of various hosts, including Leguminosae, Solanaceae, and Cruciferae. SsCTP1 might inhibit chitin-induced immunity by interacting with the conserved chloroplast proteins CPX and SKL2 in plants, thereby promoting infection in various hosts, whereas NbCPX negatively regulates the resistance of *N. benthamiana* to *S. sclerotiorum*.

## 2. Results

### 2.1. SsCTP1, a Chloroplast-Targeted Secretory Protein, Is an Important Virulence Factor of S. sclerotiorum

**Figure 1:**
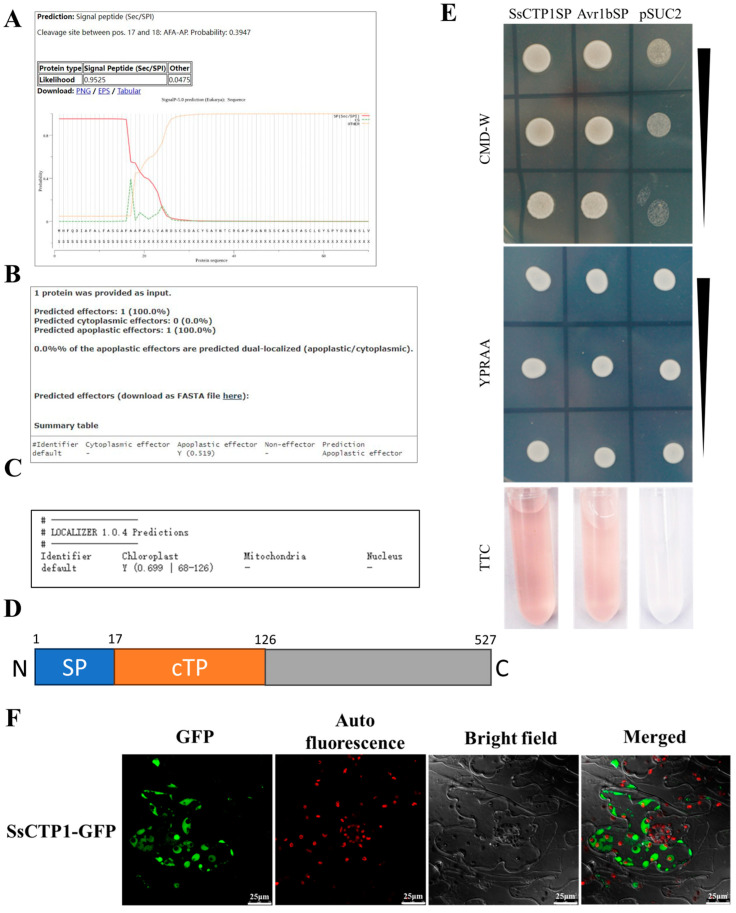
SsCTP1 is a chloroplast-targeted secretory protein. (**A**–**C**) Images taken using secretory signal peptide prediction and effector prediction pipeline analysis SS1G_13732. (**D**) A sketch of containing each designated area of SS1G_13732. (**E**) Yeast invertase secretion assay to functionally validate the predicted SP in SS1G_13732, using Avr1bSP and pSUC2 empty vectors as positive and negative controls. (**F**) Subcellular localization of proteins was observed by confocal microscopy in *N. benthamiana*. Fluorescence of GFP and chloroplast autofluorescence was monitored at 48 hpi, Scale bars, 25 μm.

When the proteins of *S. sclerotiorum* were analyzed using secretory signal peptide prediction and effector prediction pipeline analysis, we noticed that *SS1G_13732* encoded a candidate effector with a signal peptide (SP) and a chloroplast transit peptide (cTP) (Figure 1A–D). We first performed a yeast invertase secretion assay to functionally validate the predicted SP in SS1G_13732 using Avr1bSP and pSUC2 empty vectors as positive and negative controls, respectively. Both SPs from SS1G_13732 and Avr1b enabled yeast growth on the YPRAA medium and exhibited a red color with 2, 3, 5-triphenyltetrazolium chloride (Figure 1E), thereby confirming the secretory function of SS1G_13732^SP^. To explore the role of SS1G_13732 in plants, we linked the C-terminal of SS1G_13732 to green fluorescent protein (GFP) and transiently expressed it in *N. benthamiana*. The fluorescence results showed that SS1G_13732-GFP was mainly located in the chloroplasts, and a part of it was located in the cytoplasm (Figure 1F). Hence, we named SS1G_13732 as Chloroplast-Targeted Protein 1 (SsCTP1), and these results indicated that SsCTP1 is a chloroplast-targeted secretory protein.

**Figure 2:**
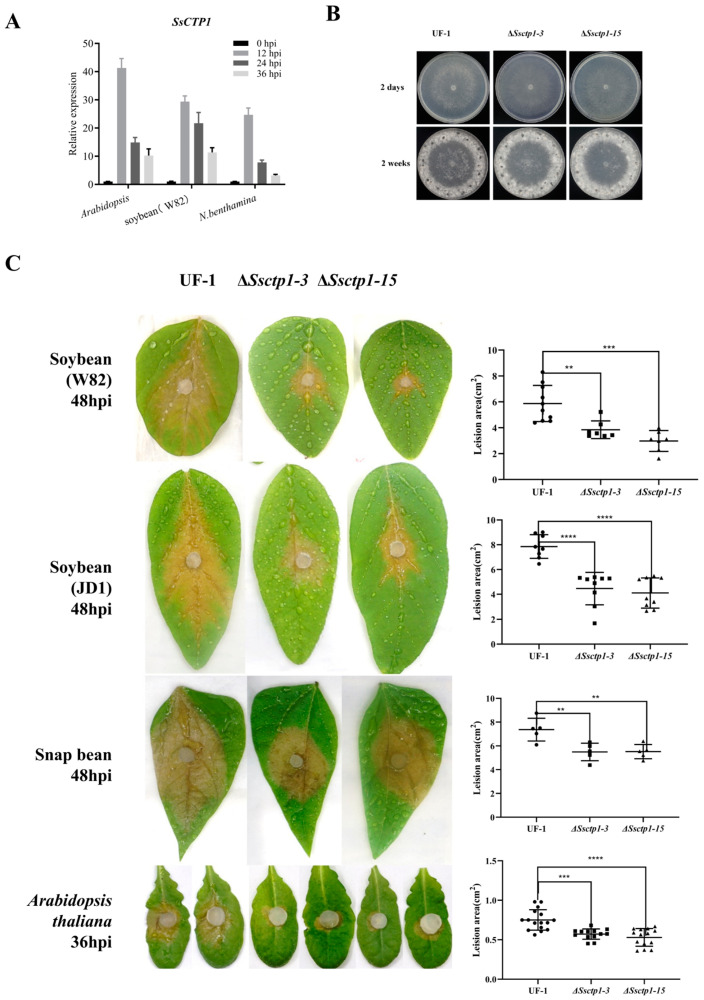
SsCTP1 is an important virulence factor in *S. sclerotiorum*. (**A**) The relative expression of *SsCTP1* was detected by way of qRT-PCR in inoculated soybeans (W82), *Arabidopsis,* and *N. benthamiana*, for 0–48 h with *Sstub1* as a reference gene for normalization. The relative levels of transcript were calculated by way of the comparative Ct method. Bars indicate ±SE. Three independent biological replicates. (**B**) Colony morphology of UF-1, Δ*Ssctp1*-3, and Δ*Ssctp1*-15 strains cultured on PDA for two days and two weeks at 25 °C. (**C**) Disease symptoms of soybean (W82, JD1, 48 hpi), snap bean (48 hpi), and *Arabidopsis* (36 hpi) leaves inoculated with UF-1, Δ*Ssctp1*-3, and Δ*Ssctp1*-15. The lesion area is measured using ImageJ 1.50i. Three independent biological replicates. **: represents significant difference from UF-1 at *p* < 0.01, ***: represents significant difference from UF-1 at *p* < 0.001, ****: represents significant difference from UF-1 at *p* < 0.0001.

To investigate the function of SsCTP1 in the pathogenesis of *S. sclerotiorum*, we first evaluated the transcriptional changes in *SsCTP1* during infection. The qRT-PCR results showed that *SsCTP1* was highly up-regulated in the early stage of infection, and its expression gradually decreased during the process of infection with *A. thaliana* (Cruciferae), soybean (Leguminosae), or *N. benthamiana* (Solanaceae) (Figure 2A). 

To explore the biological functions of SsCTP1 in *S. sclerotiorum*, two *Ssctp1* knockout mutants, Δ*Ssctp1*-3 and Δ*Ssctp1*-15, were obtained under the background of wild-type (UF-1). The colony morphology, hyphal growth rate, compound appressorium formation, and sclerotium development of the Δ*Ssctp1* mutants were not significantly different from those of UF-1 (Figure 2B), but the pathogenicity to different hosts was significantly reduced (Figure 2C). In summary, SsCTP1 is a chloroplast-targeted secretory protein that plays an important role in the pathogenesis of *S. sclerotiorum* infection in various hosts.

### 2.2. SsCTP1 Inhibits Plant Basal Immunity and Resistance to S. sclerotiorum

**Figure 3:**
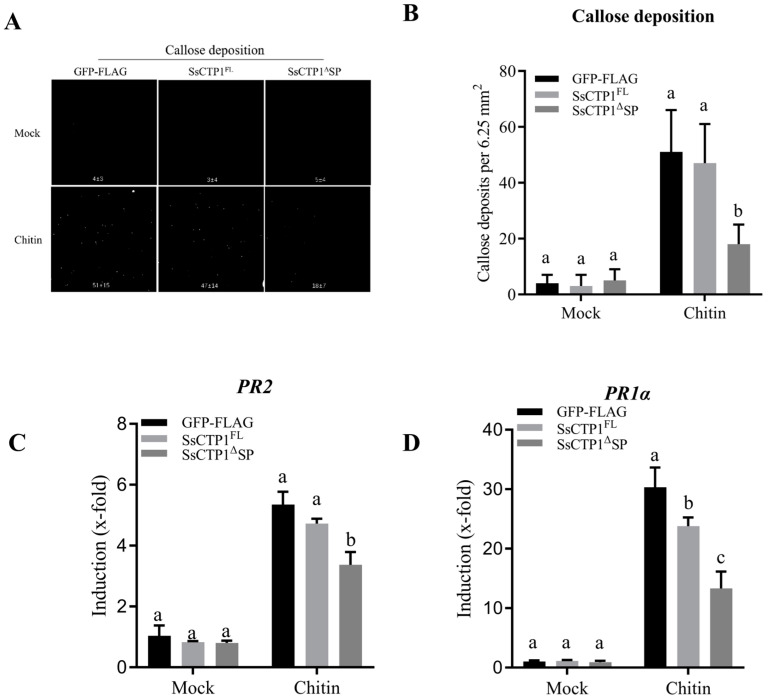
SsCTP1 inhibits plant basal immunity in *N. benthamiana.* (**A**,**B**) We observed callose deposition under a UV microscope and calculated callose deposition every 6.5 mm^2^. (**C**,**D**) We detected the expression of *PR2* and *PR1α* by qRT-PCR after chitin treatment. *NbEF-1α* used as a reference gene Three independent biological replicates. Above-mentioned, SsCTP1^FL^-FLAG, SsCTP1^ΔSP^-FLAG, or GFP-FLAG (as controls) were transiently expressed in *N. benthamiana* leaves, and leaves were infiltrated with water (Mock) or 200 µg/mL chitin, and aniline blue staining and RNA extraction were performed at 24 hpi. Different letters (a, b, c) indicate significant differences of *p* < 0.05.

**Figure 4:**
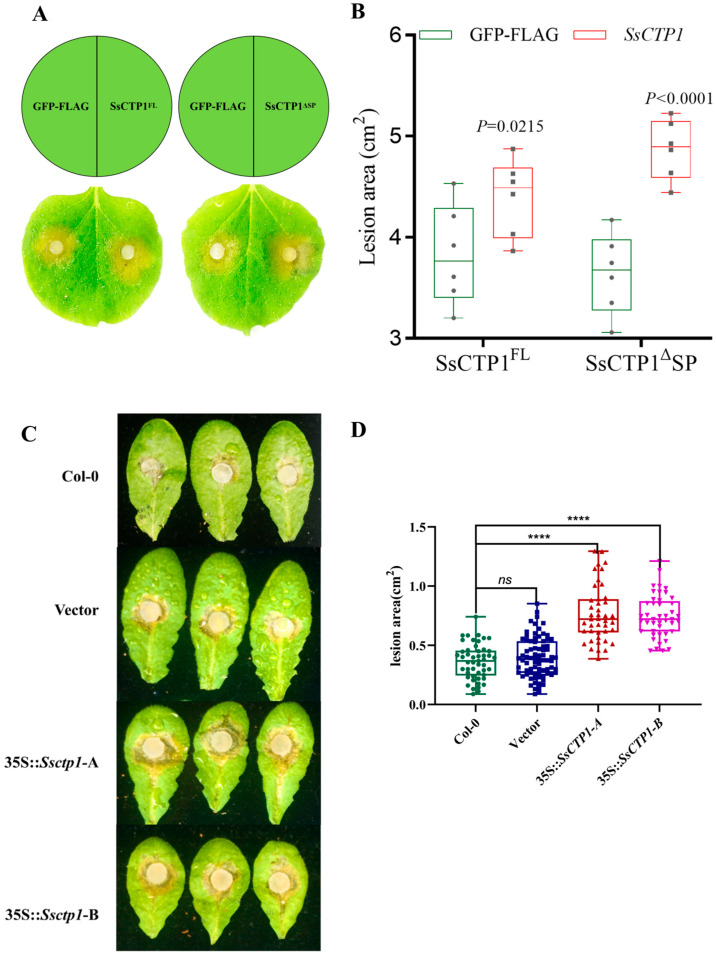
SsCTP1 inhibits plant resistance to *S. sclerotiorum.* (**A**,**B**) The transient expression of SsCTP1 in *N. benthamiana* was observed to enhance the severity of leaf disease. The photographs were taken at 24 hpi. (**B**). The lesion area is measured using ImageJ. One-way ANOVA; *n*  =  6; ****: represents significant difference from eGFP at *p* < 0.0001. The data represent means ± SD. eGFP, eGFP-FLAG as a control; SsVSPCs:SsCTP1-FL (full length) and SsCTP1-ΔSP. (**C**,**D**). Col-0, the empty vector and 35S:Ssctp1-A,35S:Ssctp1-B transgenic Arabidopsis were inoculated with *S. sclerotiorum*. The photographs were taken at 18 hpi. The lesion area is measured using ImageJ 1.50i. Statistical significance is indicated with asterisks (unpaired two-tailed Student’s *t* test, ****: represents significant difference from Col-0 at *p* < 0.0001, ns: represents no significant difference from Col-0 at *p* < 0.01). Three independent biological replicates.

To investigate the role of SsCTP1 in plant immunity, we assessed its impact of SsCTP1 on the basal immune response. The results showed that the transient expression of SsCTP1, irrespective of the presence of a signal peptide, inhibited callose deposition (Figure 3A,B) and the expression of pathogenesis-related genes (*NbPR1a*, *NbPR2*) induced by the pathogen-associated molecular patterns (PAMPs) flg22 and chitin (Figure 3C,D). Overexpression of SsCTP1 in *N. benthamiana* promoted the infection of *N. benthamiana* by *S. sclerotiorum*, suggesting that SsCTP1 suppressed basic immunity and resistance to *S. sclerotiorum* in *N. benthamiana* (Figure 4A,B).

To further study the function of SsCTP1 in *A. thaliana*, we generated transgenic *Arabidopsis* lines overexpressing SsCTP1 under the control of the 35S promoter (*35S: Ssctp1*) using the floral dip method. When inoculated with the pathogen *S. sclerotiorum* strain UF-1, the transgenic lines (*35S:Ssctp1*) exhibited larger lesion areas than the wild-type Col-0 and empty vector control (EV) lines (Figure 4C,D), indicating that the overexpression of SsCTP1 in *Arabidopsis* resulted in increased susceptibility to *S. sclerotiorum*. 

### 2.3. SsCTP1 Interacts with Chloroplast Proteins GmCPX and GmSKL2

**Figure 5:**
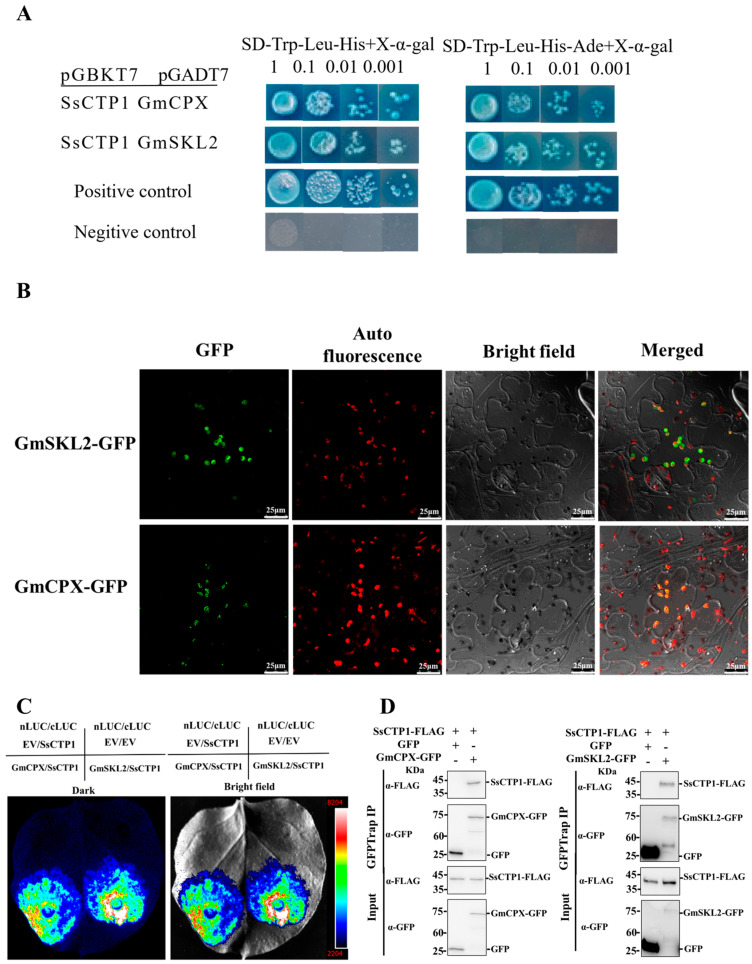
SsCTP1 interacts with the host chloroplast proteins GmCPX and GmSKL2. (**A**). Y2H technology verifies the interaction between SsCTP1 with GmCPX and GmSKL2. pGBKT7-53 + pGADT7-T is the positive control, pGBKT7-Lam + pGADT7-T is the negative control. SD-Trp-Leu-His + X-α-Gal, SD-Trp-Leu-His containing 20 mg/mL X-α-Gal. SD-Trp-Leu-His-Ade + X-α-Gal, SD-Trp-Leu-His-Ade containing 20 mg/mL X-α-Gal. (**B**) Subcellular localization of proteins was observed by confocal microscopy in *N. benthamiana*. Fluorescence of GFP and chloroplast autofluorescence was monitored at 48 hpi. Scale bars, 25 μm. (**C**) SsCTP1 interacts with GmCPX and GmSKL2 through split-luciferase complementation assays (Split-LUC). cLUC-SsCTP1 and GmCPX-nLUC, GmSKL2-nLUC were transiently expressed in *N. benthamiana* leaves. 35S:nLUC-EV and 35S:cLUC-EV were used as negative controls, respectively. Photos were captured at 48 hpi. Three repeats with similar results. (**D**) SsCTP1-FLAG and GmCPX-GFP/GmSKL2-GFP or GFP are co-infiltrated in *N. benthamiana*. The total plant proteins are extracted and incubated with GFP beads, and the target proteins are detected with anti-GFP and anti-FLAG polyclonal antibodies. Three duplicates with similar results.

To elucidate the mechanism by which SsCTP1 promotes the pathogenicity of *S. sclerotiorum*, we used SsCTP1 as bait to screen a yeast two-hybrid library derived from soybeans infected with *S. sclerotiorum*, ultimately identifying 10 interacting proteins (Table S1). The two most frequently selected proteins identified as candidate proteins were Coproporphyrinogen III oxidase (GmCPX, NP_001347283) and Shikimate kinase 2 (GmSKL2, XP_003517670). These interactions were confirmed using yeast two-hybrid assays (Figure 5A). 

Subcellular localization analysis of SsCTP1 revealed that despite containing a chloroplast transit peptide (cTP), a small portion of the protein was still localized in the cytoplasm. The subcellular localization of GmCPX and GmSKL2 showed that they were localized in the chloroplasts (Figure 5B), suggesting that SsCTP1 may have interacted with the candidate proteins within the chloroplasts.

The interaction between SsCTP1 and the candidate protein in vivo was further confirmed using the split-luciferase complementation assay (Split-LUC) and Co-Immunoprecipitation (Co-IP) (Figure 5C,D).

### 2.4. CPX Negatively Regulates the Resistance to S. sclerotiorum in N. benthamiana

**Figure 6:**
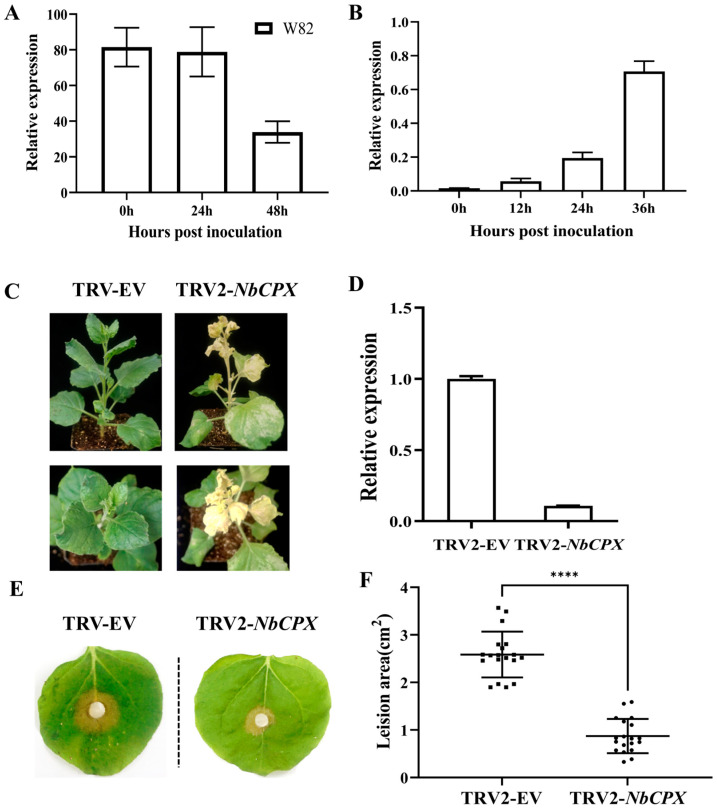
Functions of CPX. (**A**) Comparative transcriptome sequencing was analysis of GmCPX, including uninoculated WT (0 h) and WT inoculated with *S. sclerotiorum* for 24 h and 48 h (24 h and 48 h). (**B**) qRT-PCR detected the expression patterns of *NbCPX*. Plants were inoculated with UF-1 and samples were taken at different time points to detect the relative expression of genes. *GmEF-1α* as the reference gene of soybean [33]. Three biological replicates were used. (**C**) Three weeks after infiltration, *NbCPX*-silenced *N. benthamiana* plants exhibited photobleaching symptoms. (**D**) qRT-PCR results showed that *NbCPX* expression was reduced by 90% compared to that in the control (EV). (**E**). Empty vector- and *NbCPX*-silenced plants were challenged with UF-1, and the lesion area was calculated using ImageJ at 24 hpi. (**F**) We inoculated *N. benthamiana* expressing EV and NbCPX with UF-1, and counted the lesion area at 24 hpi. The lesion area was calculated by ImageJ. One-way ANOVA; **** represents a significant difference from TRV-EVs at *p* < 0.05. Data represent means ± SD.

According to the phylogenetic tree analysis, CPX and SKL2 are highly conserved across different species (Figures S1 and S2), and previous studies have suggested that CPX and SKL2 may play a role in the interaction between soybean and *S. sclerotiorum* (Figure 6A and Figure S3) [32]. To further elucidate the mechanism of action of SsCTP1, we identified the homologous protein, NbCPX, in GmCPX using BLAST. Analysis of the expression patterns of *GmCPX* and *NbCPX* revealed that the expression of *NbCPX* is up-regulated during the infection process (Figure 6B). Subsequently, we silenced *NbCPX* using a TRV-mediated gene-silencing system. Three weeks after silencing, *NbCPX*-silenced plants exhibited severe leaf chlorosis, curled tobacco leaves, and stunted growth (Figure 6C). In the silenced plants, the transcriptional level of *NbCPX* was reduced to 90% of that in the empty vector control, accompanied by mild mosaic symptoms (Figure 6D). Upon inoculation with UF-1 in vivo, the lesion area in *NbCPX*-silenced plants was smaller than that in the empty vector control, indicating enhanced resistance (Figure 6E,F). Therefore, NbCPX negatively regulates resistance in *S. sclerotiorum* in *N. benthamiana*.

## 3. Discussion

*S. sclerotiorum*, a typical broad-host-range pathogenic fungus, infects more than 600 plant species. The mechanism by which it causes disease across a wide range of hosts is not yet fully understood; however, it is believed that effectors targeting conserved host targets may contribute to its pathogenicity. SsSSVP1 targets the conserved host protein QCR8 [13], inducing host cell death, which may facilitate the ability of the fungus to infect a variety of hosts. However, the mechanisms by which *S. sclerotiorum* causes diseases in a broad range of hosts remain unclear. In this study, quantitative real-time reverse transcription polymerase chain reaction (qRT-PCR) analysis of *S. sclerotiorum* during its infection of soybean tissues demonstrated a significant up-regulation of the *SsCTP1* gene expression in the initial phases of host–pathogen interaction. Research has indicated that *N. benthamiana* plants overexpressing cyanobacterial flavodoxin reductase (pFld) significantly reduced the accumulation of chloroplastic ROS when exposed to various stressors [34], markedly enhancing their resistance to *B. cinerea*. This suggests that ROS derived from chloroplasts play an important role in plant resistance to necrotrophic pathogens. Interestingly, after chitin induction, SsCTP1^ΔSP^ can suppress callose deposition and the up-regulation of defense gene expression. This indicated that SsCTP1 suppresses immunity, suggesting that it is an effector protein. More importantly, the deletion mutant ∆*Ssctp1* showed a significant decrease in pathogenicity to the host, indicating that SsCTP1 of *S. sclerotiorum* is a virulence factor involved in pathogenesis, possibly promoting the colonization of *S. sclerotiorum* through the same mechanism when infecting different hosts. From a genetic perspective, this gene is likely an immune suppressor.

Bioinformatic analysis of SsCTP1 revealed that it possesses an N-terminal signal peptide and a chloroplast-targeting sequence, Chloroplastic Transit Peptide (cTP), making it a typical secretory protein. After transient expression, it can be localized in the chloroplasts and cytoplasm of *N. benthamiana*, indicating that the cTP of SsCTP1 is functional. Pathogens secrete effector proteins that enter host cells, disrupt cell structures, affect host growth and metabolism, suppress host immune responses, and promote pathogen colonization. Fungal effectors often have multiple host targets and pleiotropic effects. Increasing evidence suggests that chloroplasts are involved in immunity. The secreted protein SsITL from *S. sclerotiorum* binds to CAS in the chloroplasts and inhibits SA accumulation regulated by CAS, suppressing the host’s immune response [15]. In this study, observations by fluorescence confocal microscopy showed that SsCTP1, GmCPX, and GmSKL2 are all localized in the chloroplasts, but SsCTP1 is localized around the position of chlorophyll autofluorescence. Although it is localized in the chloroplasts mostly, part of SsCTP1 is localized in the cytoplasm. This may be the result of long-term “game” between plants and pathogens. SsCTP1 localized in the chloroplasts directly acts with host proteins targeting the chloroplasts to suppress host immunity, and plants have evolved special mechanisms for their own growth and development, “blurring” the function of cTP to reduce the targeting of SsCTP1 to the chloroplasts. Subsequent studies will observe the interaction sites by co-localization and the distribution of SsCTP1 in the cytoplasm and chloroplasts by chloroplast separation to further reveal the interaction mechanism between SsCTP1 and the host.

Lesion-mimicking mutants (*lmms*) are plants that spontaneously develop necrotic lesions due to cell death without any pathogen infection or abiotic stress, similar to disease symptoms or HR. Many *lmms* show an up-regulation of resistance-related genes and enhance pathogen resistance. A lesion-mimicking mutant (*lin2*) isolated from *A. thaliana* forms lesions on the leaves and siliques in a developmentally regulated and light-dependent manner. Studies have shown that *LIN2* encodes CPX, a key enzyme in the tetrapyrrole biosynthetic pathway [35]. The rice light-dependent leaf lesion mimic mutant 1 (*llm1*) shows lesion spots on the leaves during the tillering stage. The formation of lesion spots in the *lmm1* mutant is attributed to programmed cell death and ROS. The *lmm1* mutant shows enhanced resistance to bacterial wilt pathogens and increased expression of PRs [23,24]. The disruption of CPX leads to the formation of necrotic spots, and *GmLMM2* encodes CPX in the chloroplasts, whereas the suppression of *GmLMM2* disrupts the chloroplast structure and the tetrapyrrole synthetic pathway. This study demonstrated the in vivo and in vitro interactions of SsCTP1 and GmCPX using Y2H, Spilt-LUC, and Co-IP techniques. To further explore the function of SsCTP1 in targeting NbCPX, the *NbCPX* gene in *N. benthamiana* was silenced using a TRV-mediated gene silencing system, and the qRT-PCR results showed that the gene silencing efficiency reached 90%. The silenced plants had severely bleached leaves, curled tobacco leaves, and a dwarf stature. This hindered the pathogenicity analysis. Subsequent studies will reduce its silencing efficiency, inoculate it with *S. sclerotiorum*, and analyze the function of this gene in the interaction between *N. benthamiana* and *S. sclerotiorum*. We further clarified the role of CPX in the molecular mechanisms underlying cell death and defense responses in soybeans. However, the precise mechanisms by which CPX functions remain unclear, and further investigation is needed to elucidate the roles of tetrapyrroles and CPX in the defense against *S. sclerotiorum*. SKL1 is a small molecule kinase. SKL1 is a regulatory factor of these processes and directly participates in the biosynthetic pathway related to the retrograde signaling pathway; in *A. thaliana*, SKL1 affects the biogenesis of chloroplasts by regulating the auxin pathway [30]. In rice, OsSKL2 can interact with OsASR1, and together, they act as important regulatory factors, conferring oxidative stress resistance as well as salt and drought tolerance by scavenging ROS. Three shikimate kinases in rice (OsSK1, OsSK2, and OsSK3) were differentially expressed during panicle development, and the expression of OsSK1 and OsSK3 was up-regulated during the heading stage after induction by N-acetyl chitosan. Plant SK may play an important role in controlling metabolic flux through the shikimate pathway, which is involved in defense responses and floral organ development. However, the mechanism of action of this gene in plant immunity has not yet been elucidated. Owing to the complexity of genetics, the immune response mechanisms of crops to many important necrotrophic pathogens and genetic regulatory factors have not been fully studied. Key necrotrophic virulence effectors are slowly emerging; however, establishing the interactions between effectors and virulence targets will improve our understanding of pathogen virulence and resistance mechanisms. Many fungal pathogens secrete specific effectors that contain the same LysM domain as the plant chitin receptor and can compete with the plant chitin receptor to bind chitin, thereby blocking the chitin-induced immune response [36]. However, it remains unclear whether *S. sclerotiorum* suppresses chitin-induced basal immunity. In the present study, we found that SsCTP1 inhibited chitin-induced callose deposition. However, it remains to be elucidated whether SsCTP1 suppresses chitin immunity by targeting CPX and SKL2 and whether the specific mechanisms are unclear and require further analysis.

## 4. Materials and Methods

### 4.1. Fungal Strains and Plant Culture Conditions

The *S. sclerotiorum* wild-type (WT) strain UF-1 was used as the experimental strain. UF-1 was maintained in potato dextrose agar (PDA) medium, and the knockout mutants were cultured on PDA medium supplemented with 100 mg/L hygromycin B. The plants (*Arabidopsis, N. benthamiana, Glycine max*.) were cultivated in a greenhouse with a photoperiod of 16/8 h condition at 22 °C. For the inoculation experiments, except for the 0.5 cm diameter mycelia plugs taken from fresh mycelia growing on PDA plates for the inoculation of A. thaliana, the other 0.7 cm diameter mycelia plugs were used. Further, 5-week-old plants were used for the pathogenicity experiments [37], and the inoculated leaves were incubated at 100% RH before the lesions were measured. 

The transgenic *Arabidopsis* plants were generated using floral dip [38]. *SsCTP1^ΔSP^* was cloned into the p3301GFP vector and transformed into *Agrobacterium tumefaciens* GV3101. The culture was shaken, collected, and resuspended in 1/2 MS medium containing AS (0.1 mM) and Silwet L-77 (0.05%). The flowering *Arabidopsis* was selected, the pods were cut off, all the inflorescences were soaked in the cultures for 1 min, the liquid was wiped off, they were horizontally placed, light was avoided overnight, and they were moved to a greenhouse. They were infiltrated again after 4–7 days. After surface sterilization, the harvested seeds T_0_ were screened on 1/2 MS medium containing 6 mg/L Basta, positive plants were transplanted into the soil, and T_1_ seeds were harvested individually. T_1_ seeds were verified if trait separation occurred, and homozygous transgenic lines were selected for the experiment. All the primers are listed in Table S2.

### 4.2. Prediction of Candidate Effector

Signal peptides (SPs) were predicted using SignalP-5.0 (http://www.cbs.dtu.dk/services/SignalP) (accessed on 16 July 2021). Whether the secreted protein is an effector protein was predicted using EffectorP 3.0 (https://effectorp.csiro.au/) (accessed on 16 July 2021), and its subcellular localization was predicted using LOCALIZER (https://localizer.csiro.au/) (accessed on 16 July 2021).

### 4.3. Gene Replacement

Deletion mutants of the *SsCTP1* gene of *S. sclerotiorum* were generated using a split-marker PCR-mediated homologous recombination strategy, and the protoplast transformation method was slightly modified [39,40]. Briefly, the flanking sequences FR1 and FR2 of SsCTP1 were amplified from the genomic DNA of the WT strain with SsCTP1-FP1/SsCTP1-RP1 and SsCTP1-FP2/SsCTP1-RP2, respectively, and the hygromycin phosphotransferase gene cassette was amplified from the pUCATPH vector to obtain overlapping HY and YG fragments using M13R/NLC37 and M13F/NLC38. FR1/HY and FR2/YG were used as templates and fused with SsCTP1-FP1/NLC37 and SsCTP1-RP2/NLC38 to obtain the final knockout fragments. For gene replacement, the previously described protoplast transformation method [41] was slightly modified. The fresh wild-type *S. sclerotiorum* UF-1 was cultured on YPSU liquid medium for 3 days, and then cut into small pieces and lysed in lysing enzymes from *Trichoderma harzianum* (L1412, Sigma-Aldrich) at 28 °C, 100 rpm, for approximately 3 h until there were no large hyphae. The cell sieve (40 µm) was filtered and washed with KCl and STC, and the concentration of the protoplasts was adjusted to 1 × 10^8^/mL and then introduced using the polyethylene glycol (PEG)-mediated transformation method. The transformants were purified by using hygromycin selection (100 µg/mL, Sigma-Aldrich, St. Louis, MO, USA) hyphal-tip at least five times and then were screened by PCR with primers M13F/M13R and Gene-F/R for identification.

### 4.4. RNA Extraction and Quantitative Real-Time PCR

Total RNA was extracted using a TransZol Up Plus RNA Kit (TransGen Biotech, Beijing, China) and combined with the All-in-One First-Strand cDNA Synthesis SuperMix for qPCR (TransGen Biotech, Beijing, China) for genomic DNA removal and reverse transcription. Quantitative real-time PCR was performed with a Top Green qPCR SuperMix (TransGen Biotech, Beijing, China) on StepOne Real-Time PCR System (Applied Biosystems, Foster City, CA, USA); data analysis adopts the 2^-∆∆Ct^ method [14].

### 4.5. Yeast Signal Peptide Screen 

The predicted N-terminal 17-amino acid SP sequence of SsCTP1 was fused in-frame to the invertase gene in the pSUC2 vector by gene synthesis by GenScript Biotech, Nanjing, China. The SP-containing effector Avr1b was used as a positive control as previously described. The recombinant constructs were transformed into yeast, and SP secretion was confirmed using a signal peptide secretion yeast detection kit (DZSL1561, Coolaber, Beijing, China).

### 4.6. Yeast Two-Hybrid System

A cDNA library of soybeans infected with *S. sclerotiorum* was screened using the mating method. The bait plasmid was verified to be non-toxic and non-self-activating to yeast cells, and the Y2H gold strain containing bait plasmid was combined with the Y187 strain containing the AD library at 30 °C for about 20 h at 30–50 rpm until a “Mickey head” appeared, and then we spread the liquid onto the defective medium. After culturing at 30 °C for 3–5 days, positive clones were selected and sequenced to obtain candidate proteins. The bait and prey plasmids were co-transformed into the yeast strain Y2H gold, and spread on SD-trp-leu, SD-trp-leu-his-X-α-gal, and SD-trp-leu-his-ade-X-α-gal medium to screen transformants, cultured at 30 °C for 3–5 days.

### 4.7. Transiently Expressed Protein in N. benthamiana

*A. tumefaciens* containing the target plasmids were collected and resuspended in infiltration buffer (10 mM MgCl_2_, 100 mM AS, 10 mM MES, pH 5.5), placed at 28 °C in the dark for 3 h, and injected into the fully expanded *N. benthamiana* leaves with a 1 mL syringe without a needle. After 48–72 h of infiltration, the tobacco epidermis was removed to detect the fluorescence signals. For Western blotting, the proteins were separated by SDS-PAGE (12%) and transferred to PVDF membranes. The corresponding anti-GFP (Sangon) and anti-FLAG (Sangon) antibodies were used as primary antibodies, with a secondary HRP-conjugated goat anti-rabbit IgG antibody (Sangon). CBB staining was performed to verify the equal loading.

### 4.8. Subcellular Localization

For subcellular localization, *N. benthamiana* leaves expressing GFP fused proteins at 2 dpi were imaged with Leica confocal microscope (Leica Microsystems, Heidelberg, Germany) LAS-X software using the preset settings for GFP (excitation, 488 nm; emission, 500–550 nm) and chlorophyll autofluorescence (Ex: 488 nm, Em: 630–670 nm). A laser intensity of 5% and gain of 10% were used to observe the chlorophyll autofluorescence.

### 4.9. Co-IP Assay

*A. tumefaciens* containing the target plasmids was infiltrated into *N. benthamiana*, and total plant proteins were extracted at 48–72 hpi. After incubating with Anti-GFP Affinity beads 4FF (Smart-Lifesciences, Changzhou, China) at 4 °C for 1 h, the excess proteins and unbound proteins were washed off with wash buffer (100 mM Tris-HCl (pH 8.0), 10 mM mol/L NaF, 2 mM mol/L NaVO_3_, 10 mM mol/L Na_2_MoO_4_, 10% glycerol, 0.5% NP-40, 150 mM NaCl, 1% protease inhibitor cocktail and 1 mM PMSF), and a 100 mL 1×SDS loading buffer was finally added, boiled for 10 min, and detected by Western blotting.

### 4.10. Virus-Induced Gene Silencing 

Virus-induced gene silencing assays were performed as previously described [40] with some modifications. The specific 300 bp fragment of *NbCPX* was obtained using the SGN VIGS Tool to silence *NbCPX* in *N. benthamiana* [42]. Then, *A. tumefaciens* carrying TRV1 and TRV2 derivatives were co-inoculated into 2-week-old *N. benthamiana* plants. The qRT-PCR technique was used to evaluate the silencing efficiency of the target gene, and *NbEF-1α* was used as an internal reference [43].

### 4.11. Luciferase Complementation Imaging (LCI) Assay

LCI assays were performed as previously described [44]. *A. tumefaciens* containing the target plasmids were collected and resuspended in infiltration buffer (10 mM MgCl_2_, 100 mM AS, 10 mM MES, pH 5.5) to a final concentration of OD600 = 0.5, placed at 28 °C in the dark for 3 h and injected into the fully expanded tobacco leaves with a 1 mL syringe without a needle. After 48 h of infiltration, luminescence signals were captured using a cooled low-light CCD imaging apparatus immediately after the infiltrated leaves were sprayed with luciferin (0.5 mM) 2 d after agro-infiltration.

### 4.12. Statistical Analyses

Statistical analyses were performed with Prism 8.0 software (GraphPad). The statistical analysis methods are described in the figure legends.

## Data Availability

The data presented in this study are available on request from the corresponding author.

## Supplementary Materials

The following supporting information can be downloaded at: https://www.mdpi.com/article/10.3390/plants13233430/s1, Figure S1: Phylogenetic trees were constructed by using the Maximum Likelihood method based on full-length amino acid sequences of CPX amino acid sequences in different species. Figure S2: Phylogenetic trees were constructed by using the Maximum Likelihood method based on full-length amino acid sequences of SKL2 amino acid sequences in different species. Figure S3: Comparative transcriptome sequencing was analysis of Gm SKL2, including uninoculated WT (0 h), WT inoculated with *S. sclerotiorum* for 24 h and 48 h (24 h and 48 h). Table S1: Candidate interaction protein screening yeast two-hybrid library derived from soybeans infected with *S. sclerotiorum*. Table S2: Primers used in this study.
