# Supplementary material for: A Virulence Factor from Sclerotinia sclerotiorum Targets the Host Chloroplast Proteins to Promote Infection
Source: Plants (Basel). 2024 Dec 6;13(23):3430. doi: 10.3390/plants13233430 (PMC11644427; doi:10.3390/plants13233430)
Supplement: Supplementary file 1 [file plants-13-03430-s001.zip › plants-3297443-supplementary.pdf]

Supporting Information

Article title: A virulence factor from Sclerotinia sclerotiorum targets the host chloroplast proteins to promote infection

Author: Wenjing Cui, Kunqin Xiao, Feng Yang, Kaibin Qiao, Xun Xu, Songyang Gu, Jinxin Guo, Zhuojian Song, Hongyu Pan, Fengting Wang, Yanhua Zhang, Jinliang Liu*

Fig. S1: Phylogenetic trees were constructed by using the Maximum Likelihood method based on full-length amino acid sequences of CPX amino acid sequences in different species.

Fig. S2: Phylogenetic trees were constructed by using the Maximum Likelihood method based on full-length amino acid sequences of SKL2 amino acid sequences in different species.

Fig. S3: Comparative transcriptome sequencing was analysis of Gm SKL2, including uninoculated WT (0h), WT inoculated with *S. sclerotiorum* for 24h and 48h (24h and 48h).

Table S1: Candidate interaction protein screening yeast two-hybrid library derived from soybeans infected with *S. sclerotiorum*.

Table S2: Primers used in this study.

Fig. S1

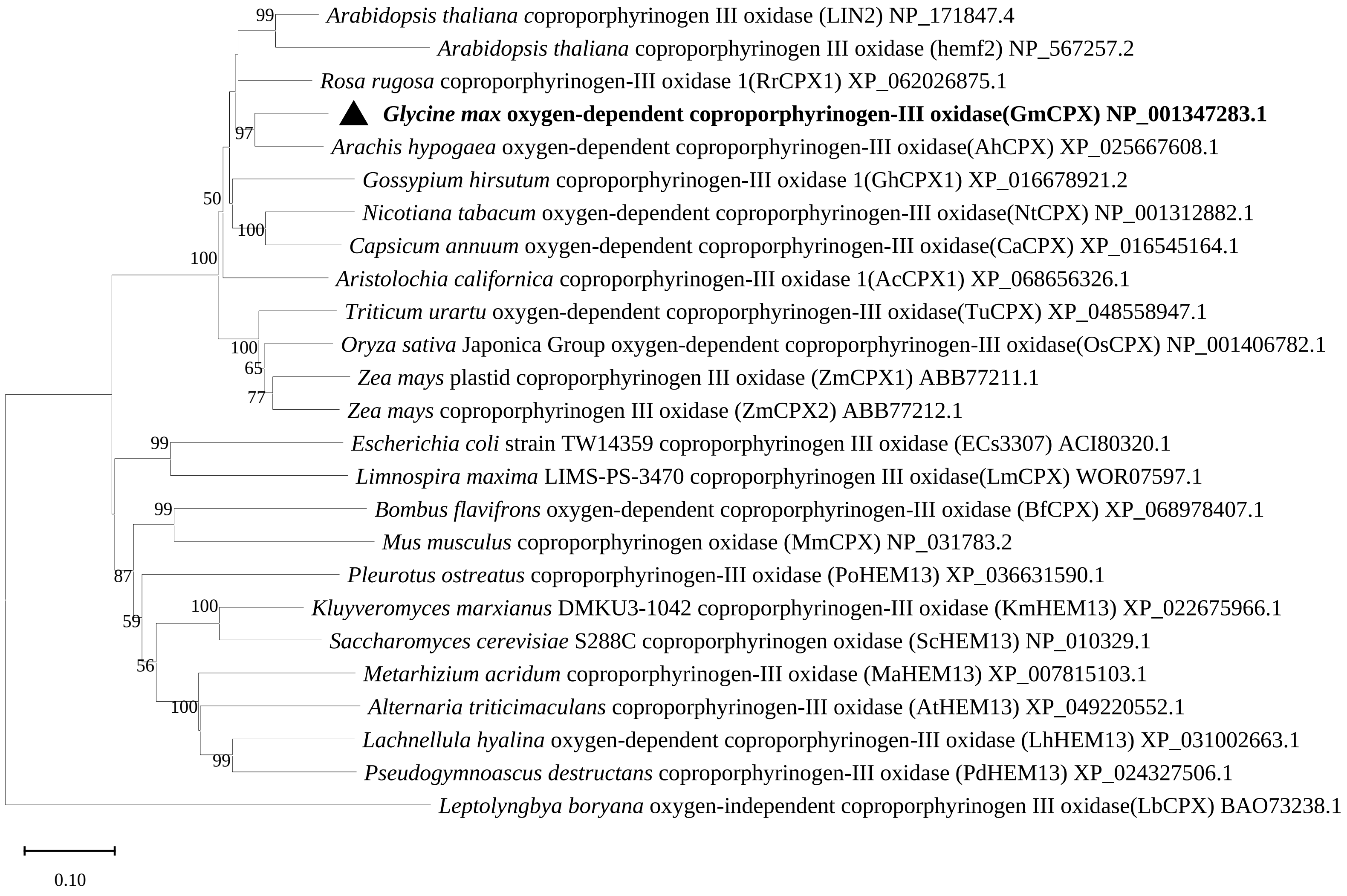
99
Arabidopsis thaliana coproporphyrinogen III oxidase (LIN2) NP_171847.4
Arabidopsis thaliana coproporphyrinogen III oxidase (hemf2) NP_567257.2
Rosa rugosa coproporphyrinogen-III oxidase 1(RrCPX1) XP_062026875.1
Glycine max oxygen-dependent coproporphyrinogen-III oxidase(GmCPX) NP_001347283.1
97
Arachis hypogaea oxygen-dependent coproporphyrinogen-III oxidase(AhCPX) XP_025667608.1
Gossypium hirsutum coproporphyrinogen-III oxidase 1(GhCPX1) XP_016678921.2
50
Nicotiana tabacum oxygen-dependent coproporphyrinogen-III oxidase(NtCPX) NP_001312882.1
100
Capsicum annuum oxygen-dependent coproporphyrinogen-III oxidase(CaCPX) XP_016545164.1
100
Aristolochia californica coproporphyrinogen-III oxidase 1(AcCPX1) XP_068656326.1
Triticum urartu oxygen-dependent coproporphyrinogen-III oxidase(TuCPX) XP_048558947.1
100
Oryza sativa Japonica Group oxygen-dependent coproporphyrinogen-III oxidase(OsCPX) NP_001406782.1
65
Zea mays plastid coproporphyrinogen III oxidase (ZmCPX1) ABB77211.1
77
Zea mays coproporphyrinogen III oxidase (ZmCPX2) ABB77212.1
99
Escherichia coli strain TW14359 coproporphyrinogen III oxidase (ECs3307) ACI80320.1
Limnospira maxima LIMS-PS-3470 coproporphyrinogen III oxidase(LmCPX) WOR07597.1
99
Bombus flavifrons oxygen-dependent coproporphyrinogen-III oxidase (BfCPX) XP_068978407.1
Mus musculus coproporphyrinogen oxidase (MmCPX) NP_031783.2
87
Pleurotus ostreatus coproporphyrinogen-III oxidase (PoHEM13) XP_036631590.1
100
Kluyveromyces marxianus DMKU3-1042 coproporphyrinogen-III oxidase (KmHEM13) XP_022675966.1
59
Saccharomyces cerevisiae S288C coproporphyrinogen oxidase (ScHEM13) NP_010329.1
56
Metarhizium acridum coproporphyrinogen-III oxidase (MaHEM13) XP_007815103.1
100
Alternaria triticimaculans coproporphyrinogen-III oxidase (AtHEM13) XP_049220552.1
Lachnellula hyalina oxygen-dependent coproporphyrinogen-III oxidase (LhHEM13) XP_031002663.1
99
Pseudogymnoascus destructans coproporphyrinogen-III oxidase (PdHEM13) XP_024327506.1
Leptolyngbya boryana oxygen-independent coproporphyrinogen III oxidase(LbCPX) BAO73238.1
0.10

Fig. S2

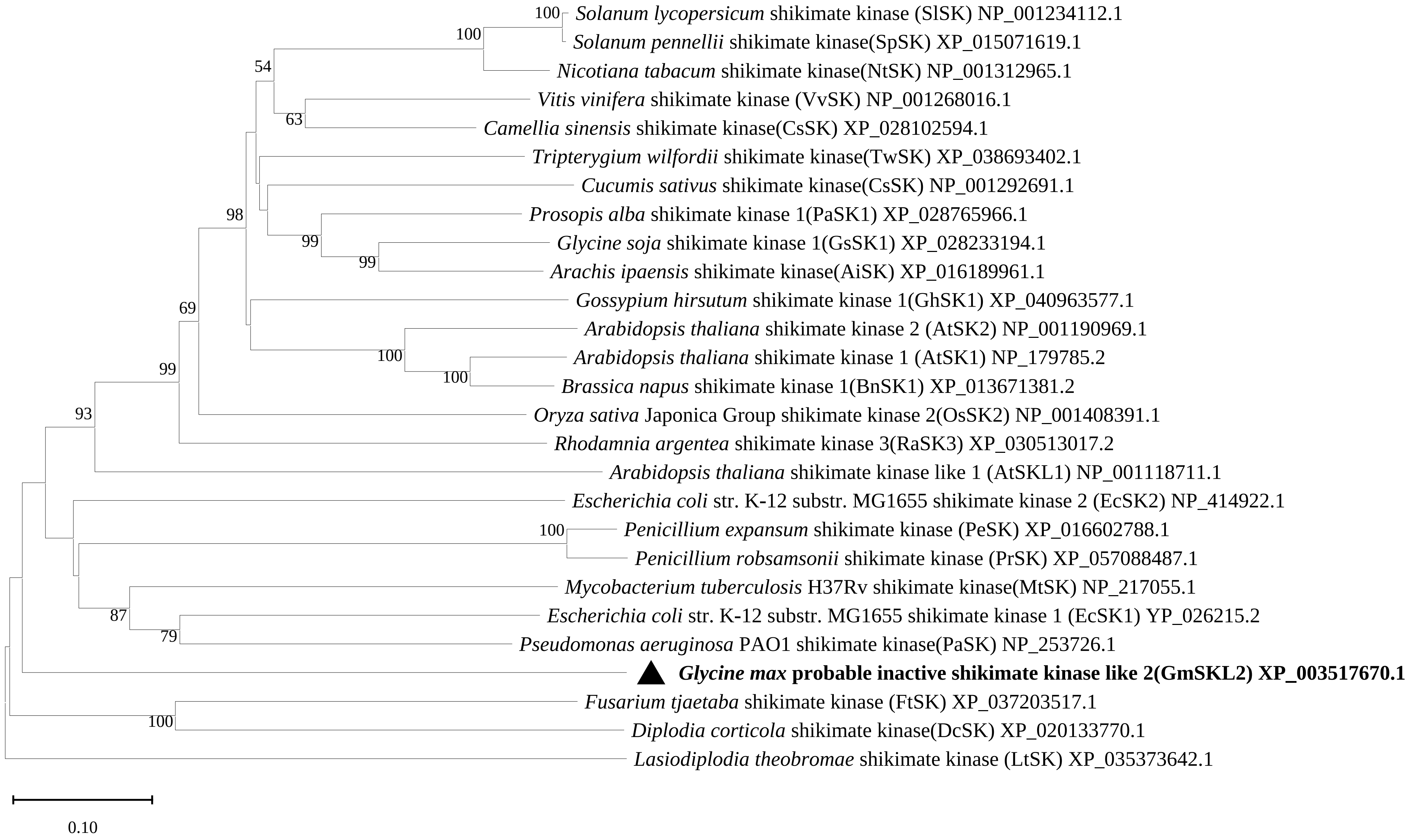
Solanum lycopersicum shikimate kinase (SlSK) NP_001234112.1
Solanum pennellii shikimate kinase(SpSK) XP_015071619.1
Nicotiana tabacum shikimate kinase(NtSK) NP_001312965.1
Vitis vinifera shikimate kinase (VvSK) NP_001268016.1
Camellia sinensis shikimate kinase(CsSK) XP_028102594.1
Tripterygium wilfordii shikimate kinase(TwSK) XP_038693402.1
Cucumis sativus shikimate kinase(CsSK) NP_001292691.1
Prosopis alba shikimate kinase 1(PaSK1) XP_028765966.1
Glycine soja shikimate kinase 1(GsSK1) XP_028233194.1
Arachis ipaensis shikimate kinase(AiSK) XP_016189961.1
Gossypium hirsutum shikimate kinase 1(GhSK1) XP_040963577.1
Arabidopsis thaliana shikimate kinase 2 (AtSK2) NP_001190969.1
Arabidopsis thaliana shikimate kinase 1 (AtSK1) NP_179785.2
Brassica napus shikimate kinase 1(BnSK1) XP_013671381.2
Oryza sativa Japonica Group shikimate kinase 2(OsSK2) NP_001408391.1
Rhodamnia argentea shikimate kinase 3(RaSK3) XP_030513017.2
Arabidopsis thaliana shikimate kinase like 1 (AtSKL1) NP_001118711.1
Escherichia coli str. K-12 substr. MG1655 shikimate kinase 2 (EcSK2) NP_414922.1
Penicillium expansum shikimate kinase (PeSK) XP_016602788.1
Penicillium robsamsonii shikimate kinase (PrSK) XP_057088487.1
Mycobacterium tuberculosis H37Rv shikimate kinase(MtSK) NP_217055.1
Escherichia coli str. K-12 substr. MG1655 shikimate kinase 1 (EcSK1) YP_026215.2
Pseudomonas aeruginosa PAO1 shikimate kinase(PaSK) NP_253726.1
Glycine max probable inactive shikimate kinase like 2(GmSKL2) XP_003517670.1
Fusarium tjaetaba shikimate kinase (FtSK) XP_037203517.1
Diplodia corticola shikimate kinase(DcSK) XP_020133770.1
Lasiodiplodia theobromae shikimate kinase (LtSK) XP_035373642.1
100
100
54
63
98
99
99
69
99
100
100
93
100
87
79
100
0.10

Fig. S3

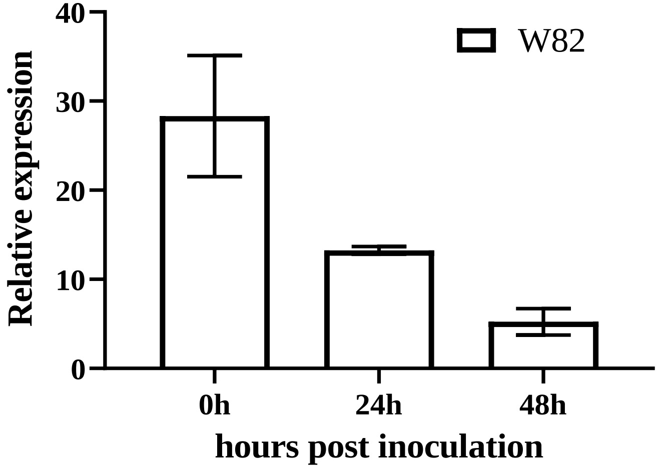

W82
40
30
20
10
0
Relative expression
0h
24h
48h
hours post inoculation

Table S1

Protein name	Locus	Start to Stop (aa)	Frequency
**isoflavone reductase**	NP_001237637	128-terminate	4/62
**MYB transcription factor MYB145**	XP_006592208	54-terminate	2/62
**chlorophyll a-b binding protein 6A**	XP_006599410.2	full-length	1/62
**eukaryotic translation initiation factor 3 subunit F**	XP_028198922	full-length	2/62
**Phosphoribulo kinase**	XP_003534305	139-terminate	2/62
**glutamine synthetase precursor**	NP_001237784.2	87-323aa	4/62
**heat shock protein 90-5**	XP_003516650	72-303	5/62
**shikimate kinase like 2, GmSKL2**	XP_003517670	81-terminate	6/62
**delta-aminolevulinic acid dehydratase**	NP_001238737.2	243-terminate	5/62
**coproporphyrinogen-III oxidase, GmCPX**	NP_001347283	226-terminate	6/62

Table S2

Primers name	Sequence 5'to 3'	Purpose
AD-F<br>AD-R	CACTACAGGGATGTTTAA<br>GGGCATTAATTCTAGTCA	yeast two-hybrid
SsCTP1-BD-F<br>SsCTP1-BD-R	GAATTCATGTACTCTCCATTCGATAACAGTGGAT<br>CTGCAGTTAGAAAGTGTAAACTGGTTGAGGA	
GmCPX-GFP-F<br>GmCPX-GFP-R	GGATCCATGATGCATTGTGCGAGCATTGTCT<br>TCTAGAGATCCATTCCTTGGGGTTGATGCAT	Co-IP assay<br>Subcellular localization of proteins
GmSKL2-GFP-F<br>GmSKL2-GFP-R	GAGCTCATGGCAACTGTTCCTGCTCGTCTCT<br>TCTAGATAACTGTGTACCAAGAGTTGTGTTA	
cLUC-SsCTP1-F<br>cLUC-SsCTP1-R	GAGCTCATGGCACCAGCTTCTTTGGTAGCTAGAG<br>GAGCTCTTAGAAAGTGTAAACTGGTTGAGGA	Spilt-LUC
GmCPX-nLUC-F<br>GmCPX-nLUC-R	GGATCCATGATGCATTGTGCGAGCATTGTCT<br>TCTAGAGATCCATTCCTTGGGGTTGATGCAT	
GmSKL2-nLUC-F<br>GmSKL2-nLUC-R	GAGCTCATGGCAACTGTTCCTGCTCGTCTCT<br>TCTAGATAACTGTGTACCAAGAGTTGTGTTA	
TRV2-NbCPX-F<br>TRV2-NbCPX-R	CTGTGAGTAAGGTTACCGAAAGAGTGTGCAAATTCCGTCA<br>GATCCATTCTTTAGGGTTGATACAATACCGGATCCCCATGGAGGC	Virus-induced gene silencing
Q-NbCPX1-F	GTGCCTACAACACATGTCGC	quantitative real-time PCR

Q-NBCPX1-R	CGCATTTGGAGCAGTTCGAC	
Q-NbEF1α-F<br>Q-NbEF1α-R	GGTTAAGATGATGCCGACCAAG<br>CGCCAGTTGGGTCCTTCTTG	
Q-NbPR1a-F<br>Q-NbPR1a-R	CCGCCTTCCCTCAACTCAAC<br>GCACAACCAAGACGTACTGAG	
Q-NbPR2-F<br>Q-NbPR2-R	AGGTGTTTGCTATGGAATGC<br>TCTGTACCCACCATCTTGC	
